# Ethyl Pyruvate Ameliorates Experimental Autoimmune Myocarditis

**DOI:** 10.3390/biom11121768

**Published:** 2021-11-25

**Authors:** Dragica Gajić, Sanja Despotović, Ivan Koprivica, Đorđe Miljković, Tamara Saksida

**Affiliations:** 1 Department of Immunology, Institute for Biological Research “Siniša Stanković”—National Institute of Republic of Serbia, University of Belgrade, Bulevar Despota Stefana 142, 11000 Belgrade, Serbia; gajic_dragica@yahoo.com (D.G.); ivan.koprivica@yahoo.com (I.K.); cvjetica@gmail.com (T.S.); 2 Faculty of Medicine, Institute of Histology and Embryology, University of Belgrade, Dr Subotića 9, 11000 Belgrade, Serbia; sanjadesp@gmail.com

**Keywords:** myocarditis, ethyl pyruvate, inflammation, interleukin-17, interferon-gamma

## Abstract

Ethyl pyruvate (EP) has profound anti-inflammatory and immunomodulatory properties. Here, its effects were determined on experimental autoimmune myocarditis (EAM) induced in mice by heart-specific myosin-alpha heavy chain peptide immunization. EP was applied intraperitoneally, daily, starting with the immunization. Severity of EAM was determined by histological assessment of immune cell infiltrates into the heart. Cells were phenotypically characterized by flow cytometry. Concentration of cytokines in cell culture supernatants and sera was determined by ELISA. EP reduced the infiltration of immune cells into the heart and lessened heart inflammation. Smaller number of total immune cells, as well as of CD11b^+^ and CD11c^+^ cells were isolated from the hearts of EP-treated mice. A reduced number of antigen-presenting cells, detected by anti-CD11c, MHC class II and CD86 antibodies, as well as of T helper (Th)1 and Th17 cells, detected by anti-CD4, IFN-γ and IL-17 antibodies, was determined in mediastinal lymph nodes draining the heart, in parallel. In the spleen, only the number of CD11c^+^ cells were reduced, but not of the other examined populations, thus implying limited systemic effect of EP. Reduced production of IFN-γ and IL-17 by myosin-alpha heavy chain peptide-restimulated cells of the lymph nodes draining the site of immunization was observed in EP-treated mice. Our results clearly imply that EP restrains autoimmunity in EAM. Therapeutic application of EP in the treatment of myocarditis in humans should be addressed in the forthcoming studies.

## 1. Introduction

Myocarditis is the inflammation of the heart muscular tissue (myocardium). Acute inflammation of the myocardium may advance to a chronic stage and cause heart tissue remodeling, fibrosis and loss of function, i.e., to the development of dilated cardiomyopathy [1]. This is a life-threatening condition, as the dilatation of heart chambers leads to systolic function impairment and heart failure, for which the only definitive treatment is organ transplantation [1]. It is assumed that up to 50% of dilated cardiomyopathy cases develop as a consequence of chronic inflammation associated with a persistent autoimmune response against the heart [1]. Experimental autoimmune myocarditis (EAM) is an important animal model of myocarditis [1]. The pathogenesis of EAM and myocarditis involves multiple arms of the innate and adaptive immune response, with CD4^+^ T cells, i.e., T helper (Th) cells and macrophages playing the dominant pathogenic role, and regulatory T cells (Treg) having a major anti-inflammatory role [1].

Importantly, elevated systemic and local levels of high mobility group box 1 protein (HMGB1) were observed in patients with myocarditis, as well as in EAM [2]. HMGB1 is a damage-associated molecular pattern that acts through the receptor for advanced glycation end products (RAGE), Toll-like receptors (TLR), and other receptors to initiate and mediate inflammation [3]. Anti-HMGB1 blocking antibodies, HMGB1 silencing by an adenoviral vector carrying shRNA, or glycyrrhizin, an HMGB1 inhibitor, were all shown protective in EAM [2,4,5]. The protective effect of HMGB1 inhibition was associated with reduced Th17 activity and reprograming of pro-inflammatory M1 macrophages in EAM [4,5,6]. Similarly, HMGB1 inhibition and reprogramming of M1 macrophages were associated with amelioration of EAM by gallein, a small molecule Gβγ inhibitor [7].

Ethyl pyruvate (EP) is a well-known inhibitor of HMGB1 [8,9]. It was previously shown to be efficient in the treatment of autoimmune and chronic inflammatory diseases, including experimental autoimmune encephalomyelitis (EAE) [10], experimental autoimmune orchitis [11], experimental colitis [12], type 1 diabetes (T1D) [13], and murine systemic lupus erythematosus [14]. These facts inspired our study of the effects of EP on EAM. We focused our investigation on the influence of EP on immune cells in the lymph nodes relevant for the pathogenesis of EAM, as well as in the heart as the target organ. Specifically, the aim of this study was to investigate phenotypic and functional properties of Th cells, Treg, and antigen-presenting cells in EP-treated EAM mice in comparison to vehicle-treated EAM mice.

## 2. Materials and Methods

### 2.1. EAM

Balb/c mice were bred and maintained in the animal facility of the Institute for Biological Research “Siniša Stanković”. The study was conducted according to the guidelines of the Directive 2010/63/EU on the protection of animals used for scientific purposes, and it was approved by the Veterinary Administration, Ministry of Agriculture, Forestry and Water Management, Republic of Serbia (No 323-07-02501/2020-05). EAM was induced in 6–8 weeks old male Balb/c mice, as previously described in detail [15]. The mice were injected with heart-specific myosin-alpha heavy chain peptide (Ac-HN-SLKLMATLFSTYASAD-OH) commercially synthesized by Bio-Synthesis Inc. (Lewisville, TX, USA) emulsified in complete Freund’s adjuvant (CFA). The peptide was dissolved in PBS to 4 mg/mL. CFA was prepared from incomplete Freund’s adjuvant supplemented with 2 mg/mL of M. tuberculosis H37Ra (both from Difco, Detroit, MI, USA). Equal volumes of the peptide solution and CFA were mixed and 100 μl of the obtained emulsion, containing 200 μg of peptide and 100 μg of M. tuberculosis, was injected subcutaneously into the posterior axillary area on days 0 and 7. Additionally, the mice were injected intraperitoneally with 100 ng of pertussis toxin on hours 0 and 48 (Tocris Bioscience, Bristol, UK). EP (100 mg/kg bw, Sigma-Aldrich) was dissolved in Hartmann’s solution (Hemofarm A.D., Vršac, Serbia) and administered intraperitoneally, starting from day 0, daily, for 21 days. Vehicle (Hartmann’s solution) was applied in parallel to the EP-untreated counterparts.

### 2.2. Histology

EAM in mice was assessed histologically at the peak of the disease, on day 21 post initial immunization. Heart tissues were fixed in 10% neutral buffered formalin and embedded in paraffin. Serial transversal sections, 5 μm thick, through heart were made and stained with hematoxylin and eosin (HE). Care was taken that sections go through both heart ventricles. To take into account histopathological variation of distribution of inflammatory infiltrates, we analyzed 5 samples with most pronounced inflammation per animal, both in EAM and EAM+EP group. Myocarditis severity was evaluated on HE-stained sections by semiquantitative scale adopted from Savvatis K. et al. [16] and Rocha R et al. [17], which took into account three parameters: infiltration of the myocardium by inflammatory cells, fibrosis, and cardiomyocyte necrosis. For each animal, five heart sections were analyzed. Each parameter was graded on the scale from 0 to 4 (grade 0 indicated no inflammation/no fibrosis/no necrosis; grade 1-small focus of inflammatory cells in close proximity to cardiomyocytes/small focus of fibrosis/necrosis; grade 2-multiple small foci of inflammatory cells/fibrosis/necrosis; grade 3-larger foci of inflammation/fibrosis/necrosis covering area around 10% of section; grade 4-severe, diffuse inflammation/fibrosis/necrosis involving more than 10% of cross section). Grading was performed by a minimum of two independent blinded investigators and averaged. The cumulative severity score was calculated as sum of infiltration, fibrosis, and necrosis severity scores.

Immunohistochemical analysis of HMGB1 expression was performed on formalin-fixed, paraffin-embedded sections using an anti-HMGB1 antibody (GT383, dilution 1:500, Thermo Fisher Scientific). After dewaxing and rehydration, a heat-inducing antigen retrieval procedure in a citrate buffer at pH 6.0 for 21 min was performed on all tissue sections, with subsequent washing in PBS and endogen peroxidase blocking with EnVisionTM FLEX Peroxidase-Blocking Reagent (SM801, Agilent Dako, santa Clara, CA, USA) for 15 min. Sections were incubated with the primary antibody for 60 min at room temperature. All sections were treated by applying the commercial EnVisionTM FLEX/HRP detection reagent (SM802, Agilent Dako). Immunoreactions were developed with diaminobenzidine (DAB, DM827 Agilent Dako) diluted in EnVisionTM FLEX Substrate Buffer (SM803, Agilent Dako). The sections were counterstained with hematoxylin. The number of HMGB1-positive cardiomyocytes was counted on randomly selected 10 fields per section, on magnification ×400. Cardiomyocytes were considered positive for HMGB1 if they contained either a nuclear or cytoplasmic HMBG1 signal. The results are presented as the percentage of HMGB1-positive cardiomyocytes.

### 2.3. Isolation of Cells and Cell Cultures

Immune cells were isolated from the hearts, which were previously perfused with PBS, on day 21 post initial immunization. Then, 70 mg of heart tissue was minced in 3.5 mL HBSS and digested with 450 U/mL collagenase I, 60 µg/mL DNase I and 60 U/mL hyaluronidase (Sigma Aldrich, Burlington, MA, USA) for 1 h at 37 °C with shaking on 320 rpm. The samples were then passed through nylon mesh (40 µm pores), centrifuged for 5 min at 550× *g*, resuspended in RPMI 5% FCS and layered upon Histopaque^®^-1077 (Sigma Aldrich). After centrifugation for 20 min on 550× *g* without rotor brakes, cells were washed twice and finally resuspended in RPMI 5% FCS for cell counting.

Cells were obtained by mechanical dissociation of mediastinal lymph nodes (MLN) or axillar lymph nodes (ALN). 

Spleen cells were obtained by mechanical dissociation followed by erythrolysis with hypotonic NH_4_Cl solution.

ALN cells were further seeded in a 96-well plate (1 × 10^6^ cells per well) and stimulated with myosin peptide (10 µg/mL) and Concanavalin A (1 µg/mL). After 24 h, cell supernatants were collected, and cytokine concentrations were determined.

### 2.4. Cytofluorometry

**Figure 1:**
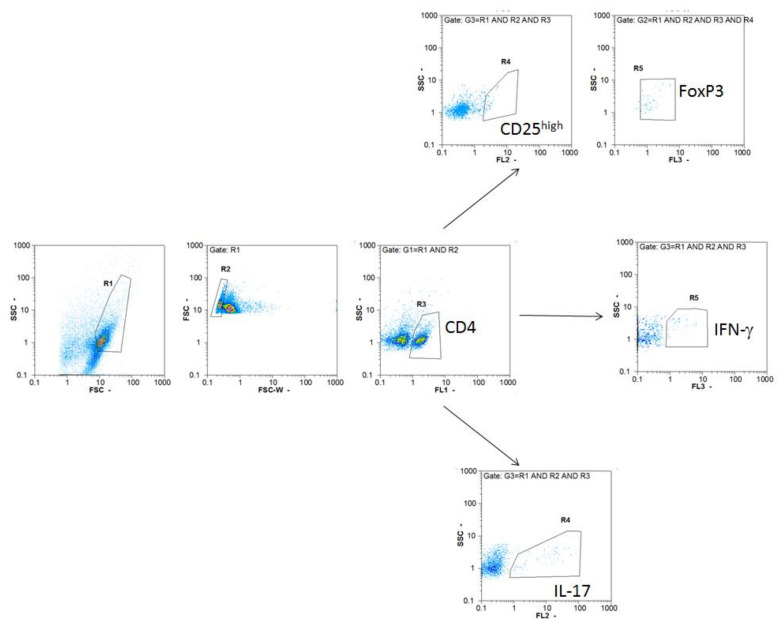
Cytofluorimetry gating strategy. Cells were gated on FSC/SSC (R1) and FSC/FSC-W (R2, single cells) and CD4^+^ cells (R3). They were additionally gated on CD25^high^ cells (R4) and FoxP3^+^ cells (R5) to determine Treg, or on IFN-γ^+^ cells (R4) to define Th1, or on IL-17^+^ cells (R4) to delineate for Th17.

Cells were stained with anti-CD4-FITC (rat IgG2b,κ), anti-CD25-PE (rat IgG1,λ), anti-CD206-PE (rat IgG2b,κ), anti-CD40-FITC (Armenian hamster IgM,κ), anti-F4/80-PerCP-Cy5.5 (rat IgG2a,κ), anti-MHC II-PE (rat IgG2b,κ), anti-CD86-PE-Cy5 (rat IgG2a,κ), anti-CD11b-PE-Cy5 (rat IgG2b,κ), anti-CD11c-PE (Armenian hamster IgG), anti-IFN-γ-PE (rat IgG1,κ), anti-IL-17-PerCP-Cy5.5 (rat IgG2a), anti-FoxP3-PE-Cy5 (rat IgG2a,κ) antibodies (all from Thermo Fisher Scientific, Waltham, MA, USA). Appropriate isotype control antibodies were used where necessary to set gates for cell marker positivity. Typically, proportion of isotype control antibody-stained cells was <1%. Cells were analyzed with a CyFlow Space flow cytometer (Partec, Munster, Germany). Results of cytofluorometry analysis are presented as proportion of cells bound by an appropriate antibody or as calculated absolute cell numbers per organ. Gating strategies for Th1, Th17 and Treg are presented in Figure 1.

### 2.5. Reverse Transcription and Real-Time PCR

Heart tissue (60 mg) was homogenized in 600 µL cold PBS. After centrifugation (20,000× *g*, 20 min) total RNA was isolated from the pellets using a mi-Total RNA Isolation Kit (Metabion, Martinsried, Germany) and reverse transcribed using random hexamer primers and MMLV (Moloney Murine Leukemia Virus) reverse transcriptase, according to the manufacturer’s instructions (Fermentas, Vilnius, Lithuania). Prepared cDNAs were amplified by using Maxima SYBR Green/ROX qPCR Master Mix (Fermentas) according to the recommendations of the manufacturer in an ABI PRISM 7000 Sequence Detection System (Applied Biosystems, Foster City, CA, USA). Thermocycler conditions comprised an initial step at 50 °C for 5 min, followed by a step at 95 °C for 10 min and a subsequent 2-step PCR program at 95 °C for 15 s and 60 °C for 60 s for 40 cycles. The PCR primers (Metabion) were as presented in Table 1.

Accumulation of PCR products was detected in real time and the results were analyzed with 7500 System Software (Thermo Fisher Scientific). Relative RNA expression is presented as 2^−dCt^, where dCt is the difference between Ct values of a gene of interest and the endogenous control (β-actin).

### 2.6. ELISA

Cytokine concentration in cell culture supernatants or serum was determined by sandwich ELISA using MaxiSorp plates (Nunc, Rochild, Denmark). For IFN-γ and IL-17 detection, anti-cytokine paired antibodies were used according to the manufacturer’s instructions (Thermo Fisher Scientific). The antibodies were as follows: anti-mouse IFN-γ purified rat monoclonal (AN-18), anti-mouse IFN-γ biotinylated rat monoclonal (R4-6A2), anti-mouse/rat IL-17A purified rat monoclonal (eBio17CK15A5) and anti-mouse/rat IL-17A biotinylated rat monoclonal (eBio17B7). Samples were analyzed in duplicates and the results were calculated using standard curves based on known concentrations of the recombinant murine IFN-γ and IL-17 (Thermo Fisher Scientific).

### 2.7. Statistical Analysis

One-way ANOVA followed by Tukey’s multiple comparison test or Student’s *t*-test (two-tailed) were used as appropriate for statistical analysis using GraphPad Prism version 6.00 for Windows, (GraphPad Software, La Jolla, CA, USA). A *p*-value less than 0.05 was considered statistically significant.

## 3. Results

### 3.1. EP Reduces Myocardial Inflammation in EAM

**Figure 2:**
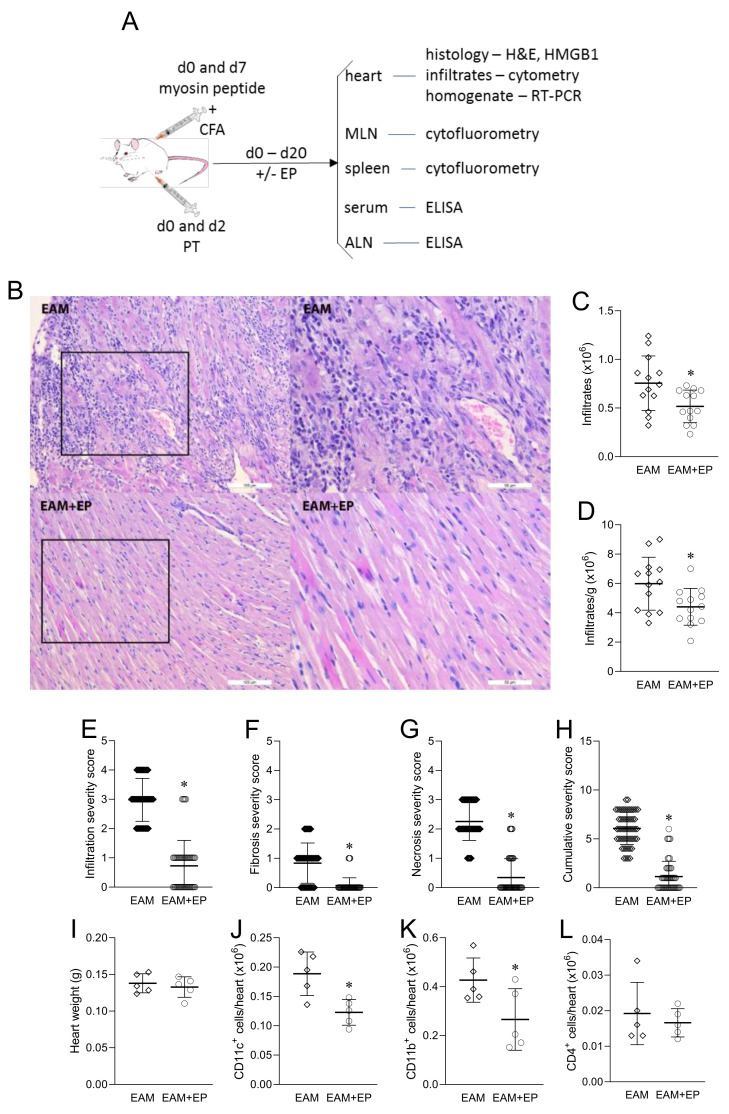
EP alleviates EAM. Balb/c mice were immunized with myosin peptide mixed with CFA on day (d) 0 and day 7. PT was applied on d0 and d2. EP (100 mg/kg) or vehicle were applied intraperitoneally, daily from d0 to d20. 21 days after the initial immunization, hearts, mediastinal lymph nodes (MLN), spleens, sera, and axillar lymph nodes (ALN) were isolated for the indicated analyses (**A**). Representative micrographs of hematoxylin- and eosin-stained heart sections are presented. Scale bar 100 µm/ 200× magnification (images on the left) and scale bar 50 µm/400× magnification (images on the right) (**B**). Absolute number of cells isolated from the hearts (**C**) and number of isolated cells normalized to 1 g of heart tissue (**D**) are presented as mean +/− SD obtained from 13 mice per group. Infiltration severity score (s.c.) (**E**), fibrosis s.c. (**F**), necrosis s.c. (**G**), and cumulative s.c. (**H**) are presented as mean +/− SD obtained from 55 sections per group. Heart weight (**I**), number of CD11c^+^ cells (**J**), CD11b^+^ cells (**K**), and CD4^+^ cells (**L**) in the heart infiltrates are presented as mean +/− SD obtained from 5 mice per group. * *p* < 0.05 EAM (rhombi) vs. EAM+EP (circles).

The effects of EP on EAM were assessed 21 days after the immunization (Figure 2A). Mice that were treated with EP typically had scarce infiltrates, unlike their vehicle-treated counterparts that had dense inflammatory infiltrates throughout the heart sections (Figure 2B–D). The inflammatory infiltrate in both groups was predominantly lymphocytic. The infiltrates were present in all obtained sections in EAM mice, and they were diffused or multifocal throughout the myocardium. The infiltrates were sparse and focally detected through samples in EAM+EP group. EAM severity grades clearly showed that EP efficiently reduced heart inflammation, infiltration (Figure 2E), fibrosis (Figure 2F), and cardiomyocyte necrosis (Figure 2G), and the cumulative severity score (Figure 2H) was also reduced in EP-treated mice. While there were no differences in heart weights between the groups (Figure 2I), absolute numbers of heart CD11c^+^ and CD11b^+^ cells were reduced in EP-treated mice (Figure 2J,K). However, the absolute number of heart CD4^+^ T cells was without difference between the EP-treated and untreated group (Figure 2L).

### 3.2. EP Affects Immune Response in Mediastinal Lymph Nodes

**Figure 3:**
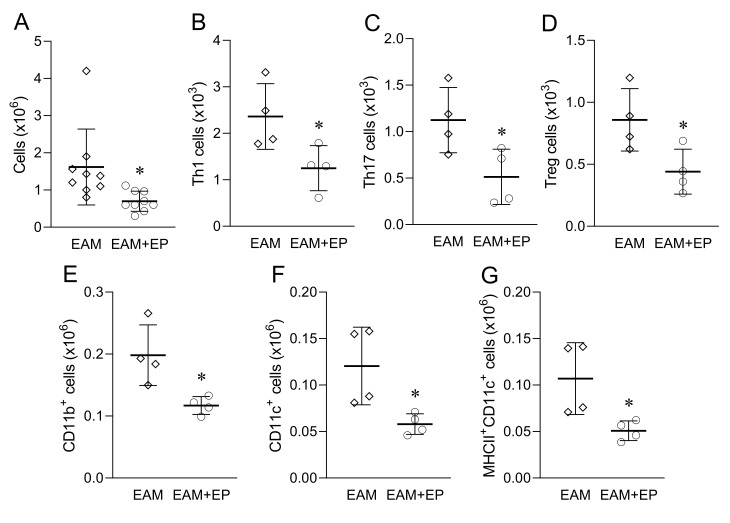
Effects of EP on MLN in EAM. Balb/c mice were treated as presented in Figure 1A. Number of cells obtained from MLN are presented as mean +/− SD from 9 mice per group (**A**). Number of Th1 cells (**B**), Th17 cells (**C**), Treg (**D**), CD11b^+^ cells (**E**), CD11c^+^ cells (**F**), and MHCII^+^CD11c^+^ cells (**G**) are presented as mean +/− SD from 4 mice per group. * *p* < 0.05 EAM (rhombi) vs. EAM+EP (circles).

The influence of EP on the immune response within MLN that drain the heart was assessed through phenotypic characterization of isolated cells. There were less cells in the mediastinal lymph nodes of EP-treated mice at day 21 post immunization (Figure 3A). Moreover, the number of Th1, Th17, Treg, CD11b^+^, CD11c^+^ cells, as well as of MHC class II-expressing CD11c^+^ cells, was also lower in the mediastinal lymph nodes of EP-treated mice (Figure 3B–F). 

### 3.3. Systemic EP Effects on the Immune Response

**Figure 4:**
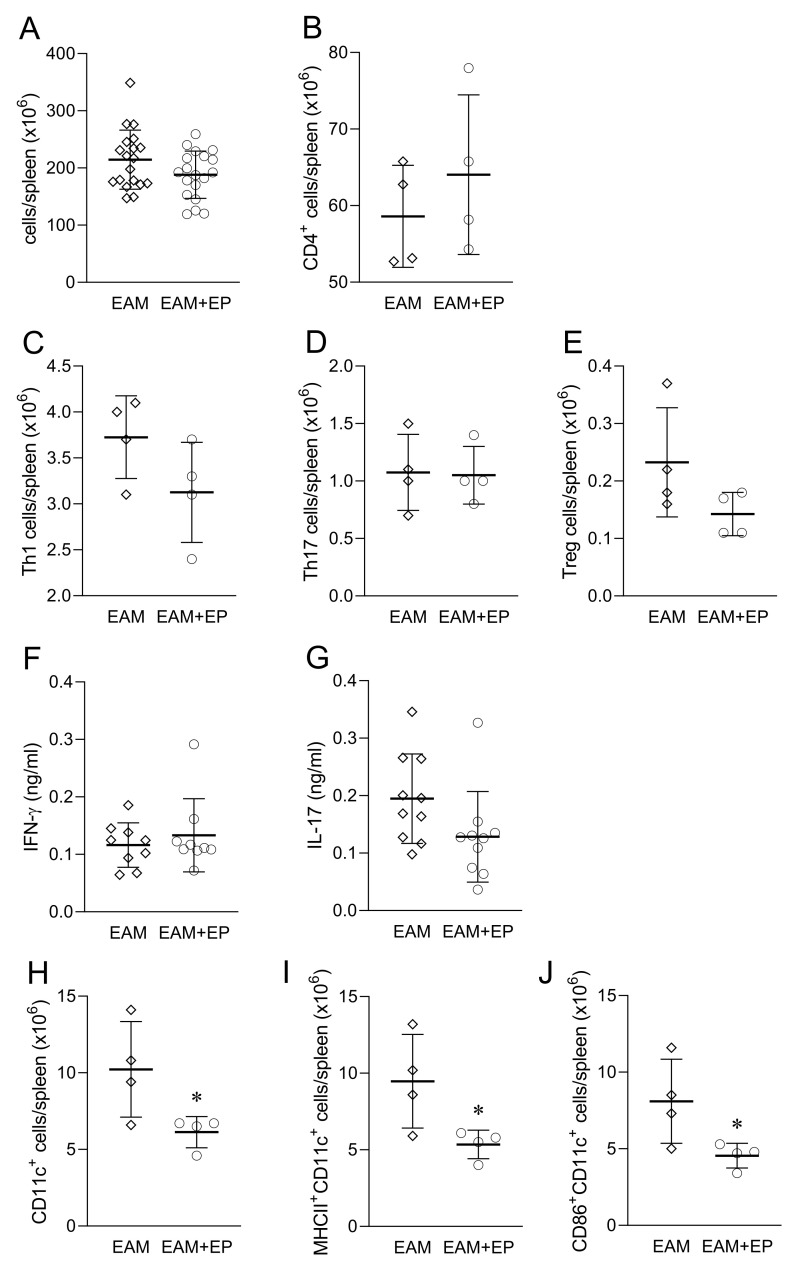
Effects of EP on spleen and serum in EAM. Balb/c mice were treated as presented in Figure 1A. Number of cells obtained from the spleen are presented as mean +/− SD from 19 mice per group (**A**). Number of CD4^+^ cells (**B**), Th1 cells (**C**), Th17 cells (**D**), Treg (**E**), CD11c+ cells (**H**), MHCII+CD11c+ cells (**I**) and CD86+CD11c+ cells (**J**) are presented as mean +/− SD from 4 mice per group. Serum levels of IFN-γ (**F**) and IL-17 (**G**) are presented as mean +/− SD from 9 mice per group * *p* < 0.05 EAM (rhombi) vs. EAM+EP (circles).

The systemic effect of EP treatment was assessed in the spleen and serum. There was no effect of EP on spleen cellularity in EAM at day 21 post immunization (Figure 4A). Absolute numbers of CD4^+^, Treg, Th1, and Th17 cells were also similar between EP-treated and vehicle-treated mice (Figure 4B–E). Accordingly, levels of IFN-γ and IL-17 were not altered in the serum of EP-treated mice (Figure 4F,G). Still, there were less CD11c^+^, CD11c^+^MHC class II^+^, and CD11c^+^CD86^+^ cells in the spleen of EP-treated mice (Figure 4H–J).

### 3.4. EP Inhibits IL-17 Release from Myosin-Restimulated Lymph Node Cells

**Figure 5:**
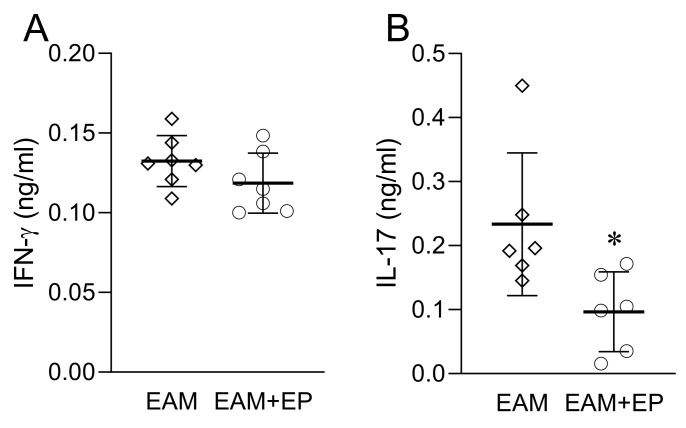
Effects of EP on ALN in EAM. Balb/c mice were treated as presented in Figure 1A. ALN cells were stimulated with myosin peptide in vitro for 24 h. Levels of IFN-γ (**A**) and IL-17 (**B**) in cell culture supernatants are presented as mean +/− SD from 6 mice per group * *p* < 0.05 EAM (rhombi) vs. EAM+EP (circles).

The influence of EP on the immune response within ALN that drain the site of immunization was also assessed at day 21 post immunization. The ALN cells were re-challenged with the myosin peptide in vitro and the level of IL-17 and IFN-γ in the cell culture supernatants was determined. While myosin-elicited production of IL-17 was lower in the ALN cells of EP-treated mice in comparison to the untreated counterparts, there was no difference between the groups in IFN-γ levels (Figure 5).

### 3.5. EP Inhibits HMGB1 and Chemokines in the Inflammed Heart

**Figure 6:**
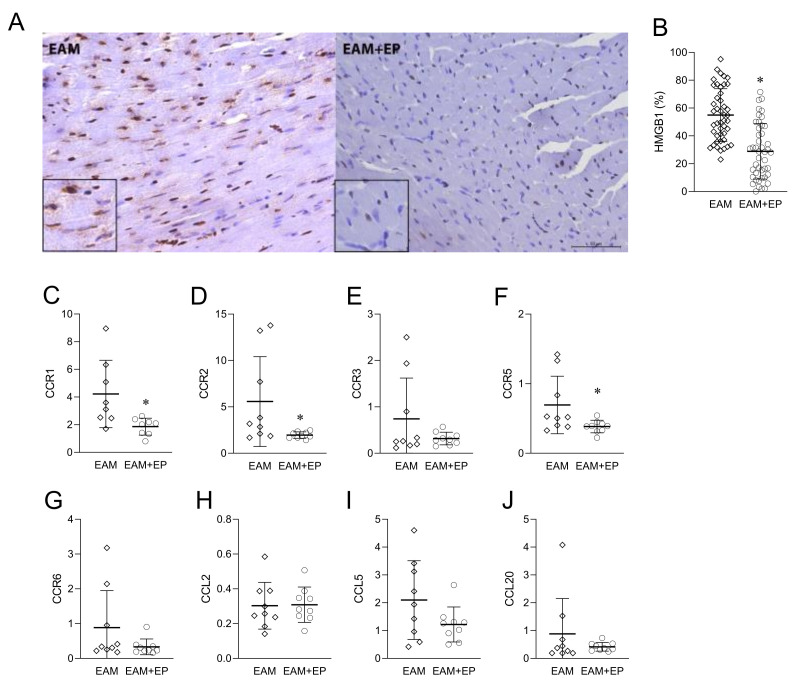
Effects of EP on HMGB1 and chemokines in the heart in EAM. Balb/c mice were treated as presented in Figure 1A. The representative images of HMGB1 staining of the myocardium in EAM and EP-treated mice are presented. Scale bar 50 µm/400× magnification (**A**). Quantification of HMGB1 staining is presented as mean +/− SD of the percentage of HMGB1-positive cardiomyocytes obtained from 46 sections per group (**B**). Expression of various chemokines and chemokine receptors was determined by RT-PCR in heart homogenates: CCR1 (**C**), CCR2 (**D**), CCR3 (**E**), CCR5 (**F**), CCR6 (**G**), CCL2 (**H**), CCL5 (**I**), and CCL20 (**J**). Data are presented as mean +/− SD from 9 mice. * *p* < 0.05 EAM (rhombi) vs. EAM+EP (circles).

HMGB1 expression in the hearts was assessed histologically 21 days post immunization. HMGB1 staining was strong in the EAM group, being dominantly present in the nuclei of cardiomyocytes (Figure 6A). Lymphocytes and endothelial cells also expressed HMGB1. In EP-treated mice, the expression of HMGB1 varied widely, with fewer positive cardiomyocytes, dominantly with nuclear positivity (Figure 6A). Quantification of HMGB1 staining showed that healthy mice had a low proportion of cardiomyocytes expressing HMGB1 (1.38+/−0.98). The percentage of HMGB1-positive cardiomyocytes was significantly reduced in EP-treated mice compared with the EAM group (Figure 6B). Expression of various chemokines and their receptors in the heart homogenates was determined by RT-PCR at day 21 post immunization. Expression of CCR1, CCR2, and CCR5 was significantly inhibited under the influence of EP (Figure 6C,D,F). Additionally, there was a trend of inhibition of CCR3, CCR6, CCL5, and CCL20 expression by EP (Figure 6E,G,I,J), while no effect was observed for CCL2 (Figure 6H).

## 4. Discussion

Our results clearly present the potency of EP to counteract the inflammatory phase of EAM pathogenesis. The beneficial effects of EP were determined in the heart, and its anti-inflammatory effects were profound in the lymph nodes draining the heart and in those draining the site of immunization. CD11c^+^ antigen-presenting cells and Th17 cells were identified as the most prominent targets of EP in EAM. 

The amelioration of EAM by EP is paralleled with the inhibition of HMGB1 expression in the heart. Thus, our results support previous findings on the beneficial effects of HMGB1-targeted interventions in EAM [2,4,5]. HMGB1 is primarily a nuclear protein, and immune cells recognize it as a damage-associated molecular pattern in the intercellular environment. Its recognition through RAGE, TLR2 and TLR4 leads to the initiation of an inflammatory response [18]. We have previously shown that the amelioration of central nervous system (CNS) autoimmunity in EAE in rats is associated with the inhibition of HMGB1 expression in the macrophages/microglia in the CNS [10,19]. Accordingly, EP was shown to be able to inhibit HMGB1 release, as well as HMGB1 phosphorylation and acetylation in rodent macrophages and microglia [20,21,22]. The beneficial effects of EP in animal models of T1D [13], rheumatoid arthritis [23], systemic lupus erythematosus [14], autoimmune orchitis [11], and inflammatory bowel disease [24,25] were also associated with HMGB1 inhibition in the tissues targeted by the pathogenesis. It is therefore justified to conclude that the inhibition of HMGB1 by EP is one of the major ways in which this compound restrains chronic inflammation and autoimmunity.

Antigen-presenting cells play an essential role in the pathogenesis of myocarditis [1]. The crucial role of CD11c^+^ dendritic cells for the presentation of cardiac self-antigens to T cells and subsequent cardiac inflammation, hypertrophy and fibrosis was shown in hemodynamic overload-induced myocarditis [26]. Additionally, CD11b deletion led to a reduction of heart damage in viral myocarditis caused by coxsackievirus B3 infection in mice, and the effect was associated with reduced Th17 activity [27]. Thus, reduction of CD11b^+^ and/or CD11c^+^ cells in EAM hearts, lymph nodes draining the heart and spleen under the influence of EP is clearly important for understanding its beneficial role in EAM. Still, CD11c^+^ dendritic cells were also demonstrated to play an immunoregulatory role in EAM, as they restrained T cell activation through NO release [28]. Nevertheless, not only the proportion of CD11c^+^ cells was reduced in EP-treated mice, but these cells also had reduced expression of MHC class II and costimulatory molecules. Previous in vitro studies clearly implied a tolerogenic effect of EP on CD11c^+^ dendritic cells, as their capacity to produce various inflammatory cytokines, present antigens, and stimulate allogeneic T cell reactivity was markedly decreased under the influence of EP [29,30]. Additionally, EP increased the abundance of tolerogenic CD11c^+^CD11b^−^CD103^+^ dendritic cells in the pancreas of T1D mice [13]. Unlike the profound effect of EP on immune cells in the heart and EAM-related lymph nodes, the effect of the compound on the immune system on a systemic level was limited. Indeed, levels of IFN-γ and IL-17 were not altered in the serum of EAM mice treated with EP at the peak of the disease. Additionally, the proportion of Th1, Th17, and Treg cells was not altered in the spleen. Still, the proportion of CD11c^+^ cells, and MHC class II^+^ or CD86^+^ cells among them were reduced under the influence of EP. This further implies that CD11c^+^ cells are particularly sensitive to the influence of EP. Still, without further studies we cannot decipher if the observed inhibition of antigen-presenting capacity in EAM under the influence of EP is the cause or the consequence of EP anti-inflammatory activity, yet we can say that the effect of EP on CD11c^+^ cells in EAM is highly associated with the reduced disease activity. Thus, the detailed investigation of the influence of EP on CD11c^+^ antigen-presenting cells in EAM is warranted.

CD4^+^ T cells can play both an inflammatory and a regulatory role in the pathogenesis of EAM, as well as of myocarditis in humans [31]. Generally, it is accepted that Th1 and Th17 cells contribute to the pathogenesis, unlike Treg that counteract inflammation and autoimmune reactivity. Th1 cells play a dominant inflammatory role in the initiation of myocarditis, yet their marker effector cytokine IFN-γ seems to be of importance for the prevention of disease progression [31]. Indeed, it was shown that IFN-γ induced the differentiation of monocytes into NO-producing dendritic cells which used NO to limit the expansion of antigen-specific T cells in EAM [28]. Our results show a lack of effect of EP on IFN-γ release from the cells of the lymph nodes draining the site of immunization which were re-challenged in vitro with the myosin peptide on day 21 after the immunization, i.e., at the peak of EAM, when the acute inflammatory phase shifts towards a chronic progressive phase [1]. Additionally, the proportion of Th1 cells in the spleen and level of IFN-γ in the serum are not affected by EP in EAM at the peak of the disease. These results imply that EP could preserve IFN-γ-associated beneficial effects in EAM. On the contrary, IL-17 level is reduced in the re-challenged lymph node cells under the influence of EP at the same time point. This is a very important effect of EP, having in mind that Th17 and IL-17 are considered crucial for the progression of EAM towards late stage dilated cardiomyopathy [31]. However, we have not followed the effects of EP on EAM after the peak of the disease, and further studies dedicated to the prolonged effects of EP in EAM are needed to reach the conclusion on the clinical importance of the observed discrepancy of the effect of EP on IFN-γ and IL-17.

Importantly, both Th1 and Th17 proportions, as well as the proportions of other immune cells are reduced in the hearts of EAM mice, thus implying that the anti-inflammatory effects of EP in the disease are, at least partially, mediated through the limitation of immune effector activity in the target tissue. Interestingly, the proportion of Treg was also reduced in EAM hearts under the influence of EP. This could be a consequence of EP-imposed limited infiltration of immune cells into the heart, and/or the limited inflammation in the heart making a robust presence of Treg in the heart unnecessary. Downregulation of chemokine receptors and chemokines expression is in line with the limited infiltration of immune cells in the heart. Elevated levels of CCR1, CCR2, and CCR5 expression in the heart of A/J mice that were immunized with cardiac troponin I to develop autoimmune myocarditis were previously reported [32,33]. Moreover, reduction of inflammation in autoimmune myocarditis by nicotine treatment also downregulated the expression of these chemokine receptors [33]. Thus, our finding of downregulated cardiac CCR1, CCR2, and CCR5 expression in EP-treated mice further supports the importance of these chemokine receptors in the pathogenesis of EAM. In addition, it implies that effects of EP in EAM are imposed through interference with inflammatory chemokine system.

Our study has some limitations that have to be stated. The major limitation of this study is that it addresses only the initial, acute phase of myocarditis, as described above. Additionally, other cytokines and relevant biomolecules, besides IFN-γ and IL-17 were not investigated. Furthermore, Th cells and Treg were not analyzed in the heart because of the low number of isolated infiltrating cells which makes the efficient intracellular detection of IFN-γ, IL-17 and FoxP3 impossible. Moreover, additional populations of immune cells, both from the innate and the adaptive arm are worthy of investigation in the heart, as well as in relevant lymphoid tissues. Methodologically, rather a low number of samples were used for analysis of the effects EP had on MLN. Still, these limitations do not jeopardize the integrity and significance of the obtained data.

In conclusion, EP is a potent modulator of EAM pathogenesis. This complements previous findings on the efficacy of EP in other autoimmune and chronic inflammatory conditions. Thus, further studies exploring the possibility of EP application in the therapy of such diseases are needed. As for EAM, it will be important to explore the prolonged effects of EP on the chronic progressive phase of the disease and its ability to counteract the development of dilated cardiomyopathy. Finally, it will be of interest to investigate EP effectiveness if applied later in the disease course.

## Figures and Tables

**Table 1:** List of primers used.

Name	Forward	Reverse
CCR1	GCAGGTGACTGAGGTGATTG	TTGGTCCACAGAGAGGAAGG
CCR2	TTTGCAACTGCCTCTTTCCT	CTTCTGTCCCTGCTTCATCC
CCR3	TCATTATTCTGGCACACAGACC	CAAGTATCACGTCCACCACCT
CCR5	CAGATGGCTTCTCCACACAA	CGGAGCTTGAGAAAAACCAG
CCR6	GGACTGGAGCTGTTCTTTGG	AGGAGGACCATGTTGTGAGG
CCL2	GCTACAAGAGGATCACCAGCAG	GTCTGGACCCATTCCTTCTTGG
CCL5	ATATGGCTCGGACACCACTC	CCTCTATCCTAGCTCATCTCCA
CCL20	GTGGGTTTCACAAGACAGATGGC	CCAGTTCTGCTTTGGATCAGCG
β-actin	CCAGCGCAGCGATATCG	GCTTCTTTGCAGCTCCTTCGT

## Data Availability

Not applicable.
